# Identifying overtraining biomarkers through proteomic analysis of extracellular vesicles derived from the central nervous system of male mice

**DOI:** 10.14814/phy2.70640

**Published:** 2025-11-02

**Authors:** Dong Heon Yi, Eun Seon Hwang, Kang Eun Ko, Tae Yeon Kim, Yun Seo Cho, Ki Hoon Yook, Adelino S. R. da Silva, Hyo Youl Moon

**Affiliations:** ^1^ Department of Physical Education Seoul National University Seoul Korea; ^2^ School of Physical Education and Sport of Ribeirão Preto University of São Paulo Ribeirão Preto São Paulo Brazil

**Keywords:** biomarker, CNS‐fatigue, extracellular vesicle, overtraining, proteomic analysis

## Abstract

Overtraining, which results in central nervous system (CNS)‐fatigue‐related symptoms, leads to a long‐term decline in performance. This study investigated CNS‐derived EV protein content under overtraining conditions to identify potential biomarkers. Eight‐week‐old C57BL/6J mice were randomly divided into three groups: sedentary (SED, *n* = 7), exercise control (EX, *n* = 8), and overtraining (OT, *n* = 9) groups. The OT group underwent an 8‐week downhill treadmill‐based overtraining induction protocol. Exercise capacity was assessed using the incremental load, exhaustion, grip strength, and rotarod tests, while motor deficits, depression and anxiety were assessed using the nest building test. Proinflammatory cytokines were measured in blood plasma, skeletal muscle, and brain tissue. CNS‐derived EVs were isolated using a two‐step EV isolation protocol. Isolated EVs underwent proteomic analyses. Mice exhibited a significant decrease in aerobic exercise capacity, high‐intensity exercise tolerance, and muscular strength. OT increased quadriceps IL‐6 and hypothalamic IL‐1β/TNFα compared to EX. Plasma IL‐2 levels tended to be higher in OT than in EX. Proteomic analysis of CNS‐derived EVs revealed a decrease in lipid metabolism‐related proteins and an increase in stress‐related proteins. Valosin‐containing proteins and catalase, which are upregulated in organs under oxidative stress, were increased. Therefore, CNS‐derived EV protein contents indicated CNS fatigue under overtraining conditions.

## 1 INTRODUCTION

The primary goal of training for all athletes is to improve their physical capacity. High‐intensity and high‐volume training programs are pivotal in inducing overcompensation, ultimately leading to improved physical performance. However, it is imperative to incorporate adequate recovery periods to facilitate training adaptations. Fatigue from overloaded training sessions may induce acute performance decrement (Halson & Jeukendrup, 2004). An imbalance between the training and recovery cycles can manifest as functional overreaching (FOR), nonfunctional overreaching (NFOR), or overtraining syndrome (Kuipers & Keizer, 1988). Meeusen et al. suggest that while FOR may cause short‐term performance decrement without serious psychological or physiological problems, recovery over several days leads to subsequent performance improvement. Athletes inadequately recovering from FOR may progress to NFOR, requiring weeks for full recovery due to psychological or physiological challenges. Prolonged exposure to such training without recovery in the NFOR state can lead to the overtraining syndrome (Kuipers & Keizer, 1988).

In addition to the decline in physical function, overtraining syndrome is typically associated with increased inflammation, negative mood and behavior, lack of motivation (Kuipers & Keizer, 1988), and poor sleep quality (Campbell et al., 2021). These symptoms can escalate into persistent dysfunctional states, leading to long‐term slumps, depression, chronic mental illness, and early retirement for athletes in various fields. Therefore, the prevention of overtraining syndrome through strategic training sessions is crucial. However, the mechanisms underlying this syndrome remain unclear despite identifying overtraining symptoms.

Researchers have explored the mechanisms and the relationship between overtraining symptoms and central nervous system (CNS) fatigue. Symptoms such as depression, linked to imbalanced serotonin and dopamine levels in the CNS (Meeusen et al., 2007), and degraded sleep quality, associated with disrupted circadian rhythm‐related genes (Zisapel, 2001), have been investigated. While the mechanisms of CNS‐related diseases are well established, there is currently no clear mechanism or reliable marker for overtraining syndrome or CNS fatigue (Anderson et al., 2021; Halson & Jeukendrup, 2004; Purvis et al., 2010).

Extracellular vesicles (EV), secreted from cells and tissues, mediate intercellular communication and reflect the originating tissue and cell conditions through miRNAs, mRNA, and proteins (Tkach & Théry, 2016). For example, EVs derived from the CNS contain markers of neuronal activity, stress response, and inflammation, such as HSP70, synaptic synapses, and glial fibrillary acidic protein (GFAP) (Kim et al., 2021). EVs have emerged as pivotal in investigating unknown biomarkers and disease mechanisms (Si et al., 2019; Tkach & Théry, 2016). Circulating EVs carry proteins and miRNAs associated with specific diseases, making them valuable in pathology research (Yuana et al., 2013). In cases of Parkinson's disease, increased α‐synuclein in CNS‐derived EVs serves as a biomarker, indicating its elevation within the brain and reflecting CNS conditions (Si et al., 2019). In the realm of exercise, EVs have been studied for their role in metabolism‐related proteins or miRNAs during physical activity (Nederveen et al., 2021; Whitham et al., 2018). Consequently, we hypothesize that these EVs may provide insight into CNS stress and dysfunction during overtraining syndrome. Furthermore, we acknowledge the crucial relationship between the CNS and overtraining.

Given the absence of a clear mechanism or biomarker for overtraining syndrome (OTS), this study aimed to investigate the effects of overtraining on the protein content of CNS‐derived EVs to establish its role as a potential biomarker of the OTS in a rodent model.

## 2 MATERIALS AND METHODS

### 2.1 Experimental animals

C57BL/6J mice (8 weeks old, male) were categorized into sedentary (SED, *n* = 7), exercise control (EX, *n* = 8), and overtraining (OT, *n* = 9) groups. The mice were kept in individual cages with controlled temperature (22 ± 2°C) with a 12:12 h light–dark cycle. The mice were provided with ad libitum tap water and a standard laboratory diet with 63.1% carbohydrate, 24.5% protein, 4.5% fat (Purina 38,057, Cargill Agri Purina Inc., Sungnam, South Korea). EX and OT underwent an 8‐week downhill treadmill protocol known to induce overtraining syndrome (Pereira et al., 2012). Forty‐eight hours before the commencement of the 8‐week exercise protocol, exhaustion velocity (EhV) was measured through the incremental loading test (ILT).

### 2.2 Nest building test

Nest‐building test commenced at 7:00 pm. Pressed cotton squares (∼3 g) were placed in each cage. After 12 h, every nest was captured before exercise. Nest‐building was scored using the Deacon method (Deacon, 2006).

### 2.3 Physical performance test

#### 2.3.1 Incremental loading test (ILT)

ILT, exhaustion test, grip strength test, and rotarod test were conducted 48 h before the commencement of the 8‐week exercise, 48 h after the last exercise session in the fourth week, and 48 h after the last exercise session in the eighth week. To mitigate acute exercise effects, the mice were euthanized 48 h after the last exercise capacity test session.

The ILT involved mice running at an intensity of 6 m/min at 0%, with 1 m/min increments every minute until failure, defined by the mice stopping at the end of the treadmill five times in 1 min. Physical prodding was used to encourage the mice. The EhV determined the intensity of the OT protocols.

#### 2.3.2 Grip strength

The grip strength test, performed 4 h after ILT, assessed muscle strength. Mice were gently held by their tails and allowed to grasp the horizontally positioned metal grid of a Grip Strength Device (Bioseb). Each mouse underwent three trials with a 3‐min rest between trials.

#### 2.3.3 Exhaustion test

Twenty‐four hours after ILT, mice ran at 36 m/min until exhaustion with physical stimulation. Exhaustion was determined when the mice stopped at the end of the treadmill five times in 1 min, and the failure time was recorded.

#### 2.3.4 Rotarod test

Motor coordination and balance were evaluated using the rotarod test (Jeong‐Do Bio & Planet Co., Ltd.). Mice were individually placed on the rotarod at an initial intensity of 10 rpm, reaching a final intensity of 40 rpm after 300 s. Mice underwent a 5 min warm‐up at 10 rpm and two main trials with a 5 min rest between trials in the cage. The latency to fall and the number of falls were recorded.

### 2.4 Exercise protocol

The 8‐week exercise program was initiated at an intensity of 60% of EhV for 15 min in the first week and increased by 15 min each week until reaching 60 min in the fourth week. After 4 weeks of exercise, ILT was conducted to determine the new EhV. The EX maintained an intensity of 60% EhV and exercised for 60 min with a flat treadmill from the fifth to the eighth week. The OT exercised at 60% of EhV for 60 min as well, but with −14% inclination, and exercised at 70% of EhV for 60 min with *a* −14% inclination at week 6. At week 7, the intensity and duration increased to 75% EhV and 75 min with *a* −14% inclination, respectively. In the eighth week, the OT performed two sessions of 75 min of exercise at 75% EhV with a 4 h rest period, with *a* −14% inclination as well. The experimental protocol was modified from (Rocha et al. 2015).

### 2.5 Blood plasma and tissue collection

For animal experiments, mice were anesthetized with isoflurane, and blood was collected via cardiac puncture using a 1 mL syringe (Sterile Hypodermic Syringe, Korea Vaccine Co., Ltd.) and transferred to an anticoagulant‐treated 3 mL tube (13 × 75 mm 3.0 mL BD Vacutainer® plastic P700 plasma tube. Lavender BD Hemogard™ closure, SKU: 366473, BD bioscience). The whole blood was centrifuged for 10 min at 1000×*g*, 4°C, and the supernatant underwent additional centrifugation for 10 min at 2000×*g*, 4°C. Isolated plasma was aliquoted into 300 μL portions for EV isolation and 10 μL aliquots for cytokine chip array.

Quadriceps and hypothalamus were removed after blood collection. The tissues were stored at −80°C for the next step.

### 2.6 CNS‐derived EV isolation

Plasma was mixed with equal distilled water (DW) and centrifuged for 10 min at 4500 × *g*, 4°C. Following the manufacturer's protocol (63 μL of Exoquick solution per 250 μL of biofluid sample), the supernatant was mixed with an Exoquick exosome precipitation kit (System Biosciences, Inc., Mountainview, CA; Cat# EXOQ20A‐1). After 1 h of incubation, samples were centrifuged for 30 min at 1500×*g* at 4°C. The pellet was resuspended in phosphate‐buffered saline (PBS) over 3 h on a rotating mixer (KBT, Korea) after gentle mixing by pipette.

The L1 cell adhesion molecule (L1CAM), a membrane protein of CNS‐derived EVs, was chosen for immunoprecipitation to isolate EVs from the CNS. An IgG isotype control (Mouse IgG2a kappa Isotype Control Biotin, eBioscience™; Cat# 13‐4724‐85) was a negative control. Fifty microliters of 3% bovine serum albumin (BSA) containing 4 μg of L1CAM antibody (CD171 Monoclonal Antibody (eBio5G3 (5G3)), Biotin, eBioscience™; Cat# 13‐1719‐82) was added to 500 μL of EV + PBS and incubated for 1 h at 4°C with gentle rotation on a mixer.

After 1 h of incubation, 25 μL of 3% BSA containing 15 μL of Streptavidin beads (Thermo Scientific™ Pierce™ Streptavidin Plus UltraLink™ Resin; Cat# 53116), was added and incubated for 30 min at 4°C with continuous mixing. Samples were centrifuged for 10 min at 200×*g*, 4°C, and the supernatant was discarded. The remaining pellet was resuspended in 200 μL of 0.1 M Glycine‐HCl pH 2.2 (0.1 M Glycine‐HCl buffer, Tech&Innovation) with 10 s of vigorous vortexing, followed by gentle rotation for 1 min and centrifuged for 10 min at 4500×*g*, 4°C to detach L1CAM + EV from the antibody‐beads complex.

One hundred microliters of 1 M Tris–HCl (Invitrogen™ UltraPure™ 1 M Tris–HCl, pH 8.0) and DW were added to the supernatants in a fresh 1.5 mL tube to normalize the pH to 7. Then, 10% of the sample was aliquoted and diluted to 1:200 in PBS for nanoparticle tracking analysis (NTA). 200 μL of M‐PER with protease inhibitor (cOmplete™ Protease Inhibitor, Roche, PHOSS‐RO; cat# 4693116001) and phosphorylation inhibitor (PhosStop™, Roche, PHOSS‐RO; cat# 4906845001) were added to the remaining 90% of the sample to lyse and extract proteins from L1CAM + EV. The final suspensions were stored at −80°C for the next step.

### 2.7 NTA

The mean diameter (nm) and concentration (particles/mL) of EV were determined using a Nanosight LM10 system with a 642 nm laser module and NTA 3.1 nanoparticle tracking software (Malvern Instruments, Malvern, UK).

### 2.8 Western blot

Samples were loaded onto a 4%–12% NuPAGE Bis‐Tris Mini gel (Invitrogen) and run at 200 V for 30 min. The iBlot™ 2 Gel Transfer Device (Invitrogen) was used for transfer following the manufacturer's protocol. Blueye Prestained Protein Ladder (Simply™) was used for IL‐6 and TNF‐α detection in hypothalamus samples, while the Precision Plus Protein™ Kaleidoscope™ Prestained Standards (Bio‐Rad) were used for all other targets.

Primary antibodies including L1CAM (CD171 Monoclonal Antibody (eBio5G3 (5G3)), Biotin, eBioscience™; Cat# 13–1719‐82), CD81 (CD81 Antibody (B‐11), Santa Cruz; Cat# sc‐166,029), NeuN (NeuN (D4G4O) XP® Rabbit mAb, Cell Signaling; Cat# 24307), CD9 (Anti‐CD9 Antibody, Mouse Monoclonal, Sino Biological Inc.; Cat# 11029‐MM01), IL‐1β (IL‐1β (3A6) Mouse mAb, Cell Signaling; Cat# 12242), IL‐6 (IL‐6 Monoclonal antibody, Biotech; Cat# 66146‐1‐Ig), TNF‐α (TNF Alpha Polyclonal antibody, Cell Signaling; Cat# 3707), GFAP (Anti‐GFAP antibody [EPR1034Y], Abcam; Cat# ab68428) and GAPDH (GAPDH (14C10) Rabbit mAb, Cell Signaling; Cat# 2118S) were used, with Streptavidin‐HRP (Invitrogen™; Cat# 434323), anti‐mouse (m‐IgGκ BP‐HRP, Santa Cruz; Cat# 516102), and anti‐rabbit (Anti‐rabbit IgG, HRP‐linked Antibody, Cell Signaling; Cat# 7074S) serving as secondary antibodies. Immune complexes were visualized using the Immobilon® Western (HPR substrate peroxide solution, Millipore; Cat# WBLUF0500), and the western blot bands were quantified using ImageJ software. Band intensities were normalized to the corresponding loading control, and relative expression levels were calculated for each sample.

### 2.9 Proteomic analysis

The Filter‐assisted sample prep (FASP) digestion section quantified protein samples using the BCA assay (BCA Protein Assay Kit, Pierce, Cat# 23225). Proteins were digested using the filter‐aided sample preparation (FASP) method. After quantification, proteins were reduced by adding 500 mM TCEP (final concentration 5 mM) and incubated at 37°C with shaking at 300 rpm for 30 min. Next, the samples were loaded onto 10 kDa molecular weight cut‐off filters and centrifuged at 14,000×*g* for 15 min at 20°C. For alkylation, 500 mM IAA (final concentration 50 mM) was added, and the samples were incubated in the dark at 25°C for 1 h before another centrifugation at 14,000×*g* for 15 min at 20°C. Then, 200 μL of 8 M urea in 0.1 M Tris/HCl (pH 8.5) was added and centrifuged (this step was repeated three times), followed by three washes with 100 μL of 50 mM ammonium bicarbonate (ABC). Trypsin (Trypsin, Promega, Cat# V5111) digestion was performed in two rounds: first, trypsin dissolved in 50 mM ABC was added at a 1:50 enzyme‐to‐protein ratio and incubated at 37°C with 300 rpm for 12 h; then, a second digestion was carried out by adding trypsin in 50 mM ABC at a 1:100 ratio, incubated at 37°C with 300 rpm for 6 h, followed by centrifugation at 14,000×*g* for 15 min at 20°C. Finally, 40 μL of 50 mM ABC was added and centrifuged twice before the final product was dried using a Speed‐vac vacuum concentrator.

Following FASP digestion, desalting was performed using C18 Micro Spin‐Columns, which involved sequential centrifugation with different solvents to remove salts. The samples were dissolved in solvent A, loaded onto the column, and centrifuged to separate the flow‐through. Solvent A was repeatedly added and removed, and finally, solvent B was used to elute the peptides. Desalted samples were then dried for storage.

For peptide and protein identification, Percolator, Peptide Validator, and Protein FDR Validator nodes were applied with a target false discovery rate (FDR) of 1% (*q*‐value ≤0.01) at the PSM, peptide, and protein levels, respectively. Only high‐confidence identifications were retained for downstream analysis.

For LC–MS/MS analysis, peptides were first loaded onto a C18 trapping column (3 μm, 75 μm × 2 cm) and then separated on a PepMap™ RSLC C18 analytical column (2 μm, 75 μm × 50 cm). Mobile phase A consisted of water with 0.1% formic acid, and mobile phase B consisted of 80% acetonitrile with 0.1% formic acid. A gradient was applied from 4% B to 40% B over 120 min, ramped to 96% B by 130 min, held briefly, and then returned to 4% B by 180 min. The flow rate was 300 nL/min, and mass spectra were acquired over an m/z range of 400–2000.

LC–MS/MS data from each sample were analyzed using Proteome Discoverer. The UniProt Mus musculus database was used for peptide identification. Modifications included acetylation, oxidation, carbamylation, and carbamidomethylation.

Percolator, Peptide Validator, and Protein FDR Validator nodes were applied with a target false discovery rate (FDR) of 1% (*q*‐value ≤0.01) at the PSM, peptide, and protein levels. Only high‐confidence identifications were retained for downstream analysis. Normalization and filtration steps were applied to minimize technical variations.

For downstream enrichment analysis, differentially expressed proteins were defined as those with a *p*‐value <0.05 and an expression ratio ≥ |2|, based on the filtered high‐confidence protein set. Enrichment analysis was performed using DAVID to identify functionally relevant biological processes and pathways (*p* < 0.05 and fold‐change ≥ |2|). Only GO terms with a *p*‐value <0.05 (Benjamini‐Hochberg corrected) were considered significant. The analysis focused on biological processes to elucidate functional categories relevant to overtraining and CNS fatigue.

The mass spectrometry proteomics data have been deposited to the ProteomeXchange Consortium via the PRIDE partner repository with the dataset identifier PXD069257.

### 2.10 Cytokine array

Samples were analyzed using a semi‐quantitative Mouse Cytokine Array GS1 (Mouse Cytokine Array GS1, RayBiotech; cat# GSM‐CYT‐1‐1), detecting 20 proteins in one experiment. Following the manufacturer's instructions, the microarray‐based antibody array was blocked, treated with samples, and incubated with a secondary biotinylated antibody mixture. After washing, Cy3‐conjugated streptavidin (1500X HiLyte Plus™ 555 Streptavidin‐Fluor, RayBiotech; cat# AA‐HRP‐G) was used for fluorescent detection. Signals were detected using a laser scanner (GenePix 4100A, Molecular Devices) and analyzed with GenePro7.0 software (Molecular Devices).

### 2.11 Statistical analysis

Statistical analyses were performed using GraphPad Prism v.9 software (GraphPad Software Inc.). All diagrams and data present mean ± standard deviation (SD). Students' *t*‐tests were conducted for the EX and OT. One‐way analysis of variance (ANOVA) identified statistically significant differences among SED, EX, and OT. A two‐way ANOVA test was performed to detect changes in physical performance throughout the weeks between the SED, EX, and OT. Bonferroni's multiple comparisons test was conducted for the post hoc test. A *p*‐value <0.05 was accepted as statistically significant.

## 3 RESULTS

### 3.1 Physical performance decrement during 8‐week overtraining

**FIGURE 1:**
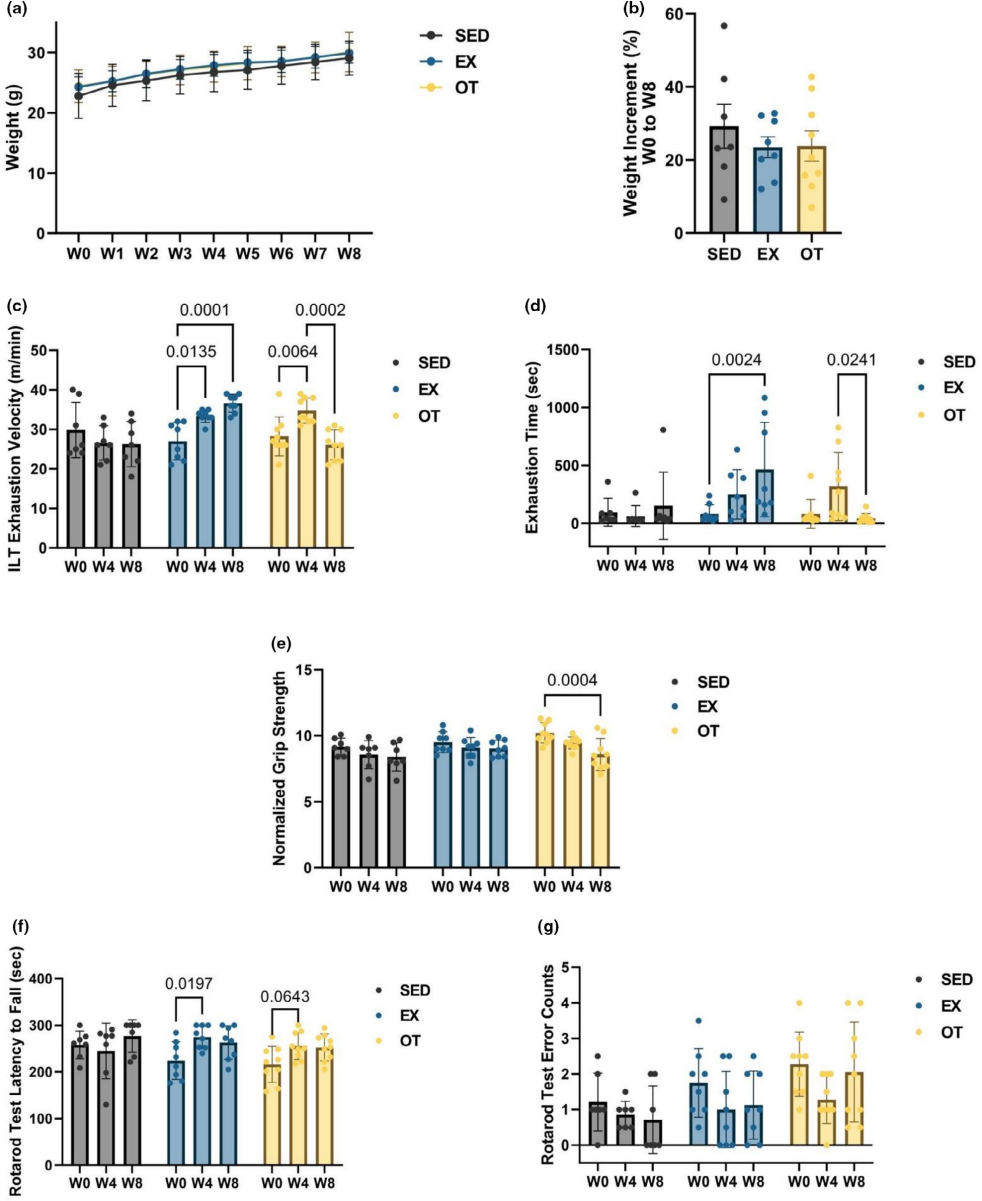
Physical performance parameters. In (a), body weight progression from W0 to W8 is depicted. The increment in body weight from W0 to W8 is illustrated in (b). In (c), the exhaustion velocity during the incremental loading test at W0, W4, and W8 is presented. The exhaustion time during the exhaustion test at W0, W4, and W8, with a 36 m/min velocity, is presented in (d). In (e), the grip strength results normalized by the corresponding week are presented. The latency to fall in the 300 s rotarod test is displayed in (f), while the error counts in the 300 s rotarod test are displayed in (g). Data for (a–g) (SED, *n* = 7, EX, *n* = 8, and OT, *n* = 9) are presented as mean ± SD. Two‐way ANOVA for (a), (c–g), and one‐way ANOVA for (b).

Figure 1a,b show that SED mice gained more body weight than EX and OT over the 8 weeks, but no statistical significance was found in any of the mice groups (Figure 1a,b). Figure 1c illustrates an increase in exhaustive velocity at ILT from W0 to W4 for both EX (*p* < 0.05) and OT (*p* < 0.01) with statistical significance (Figure 1c). After W8, EX showed a non‐statistical increase of EhV from W4 to W8, whereas the EX still increased the EhV compared to W0 at W8 (*p* < 0.001). OT significantly decreased EhV from W4 to W8 (*p* < 0.001), with a lower average than that at W0 (Figure 1c). EX significantly increased the exhaustion time at the exhaustion test from W0 to W8 (*p* < 0.01). In contrast, OT significantly decreased the exhaustion time from W4 to W8 (*p* < 0.05) (Figure 1d). SED and EX showed no differences in the normalized grip strength test between W0, W4, and W8 (Figure 1e), whereas OT showed significantly decreased results from W0 to W8 (Figure 1e; *p* < 0.001). The EX and OT had an increased latency to fall at the rotarod test from W0 to W4 (*p* < 0.05, *p* = 0.0643) (Figure 1f). The error counts at the rotarod test showed no difference among each time point in all groups (Figure 1g).

### 3.2 Overtraining influenced nest building test score

**FIGURE 2:**
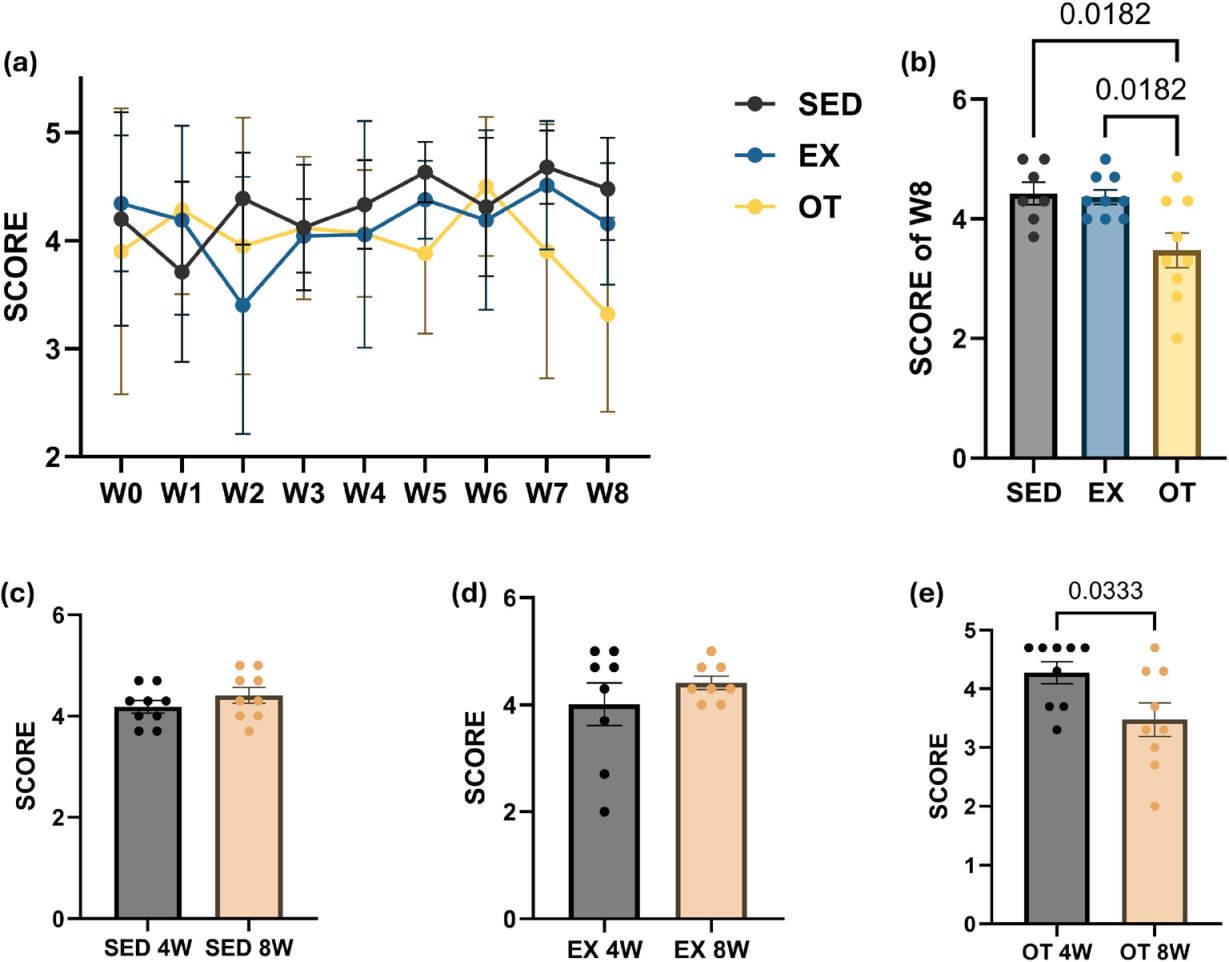
Nest building test result. (a) illustrates the nest building test score through the 8 weeks of treadmill exercise of SED, EX, and OT (b) illustrates the nest building test score at W8 of SED, EX, and OT. (c–e) illustrate the comparison of nest building test score between W4 and W8. Data for (a–e) (SED, *n* = 7, EX, *n* = 8, and OT, *n* = 9) are presented as mean ± SD. One‐way ANOVA for (b), and unpaired t‐test for (c–e).

OT significantly scored lower than SED and EX at W8 (Figure 2b; *p* < 0.05). While SED and EX have no significance from W4 and W8 (Figure 2c,d; *p* = 0.2795, *p* = 0.3537), OT has shown a significant decrease in score from W4 to W8 (Figure 2e; *p* < 0.01).

### 3.3 Overtraining induced proinflammatory cytokines in blood, quadriceps, and hypothalamus

**FIGURE 3:**
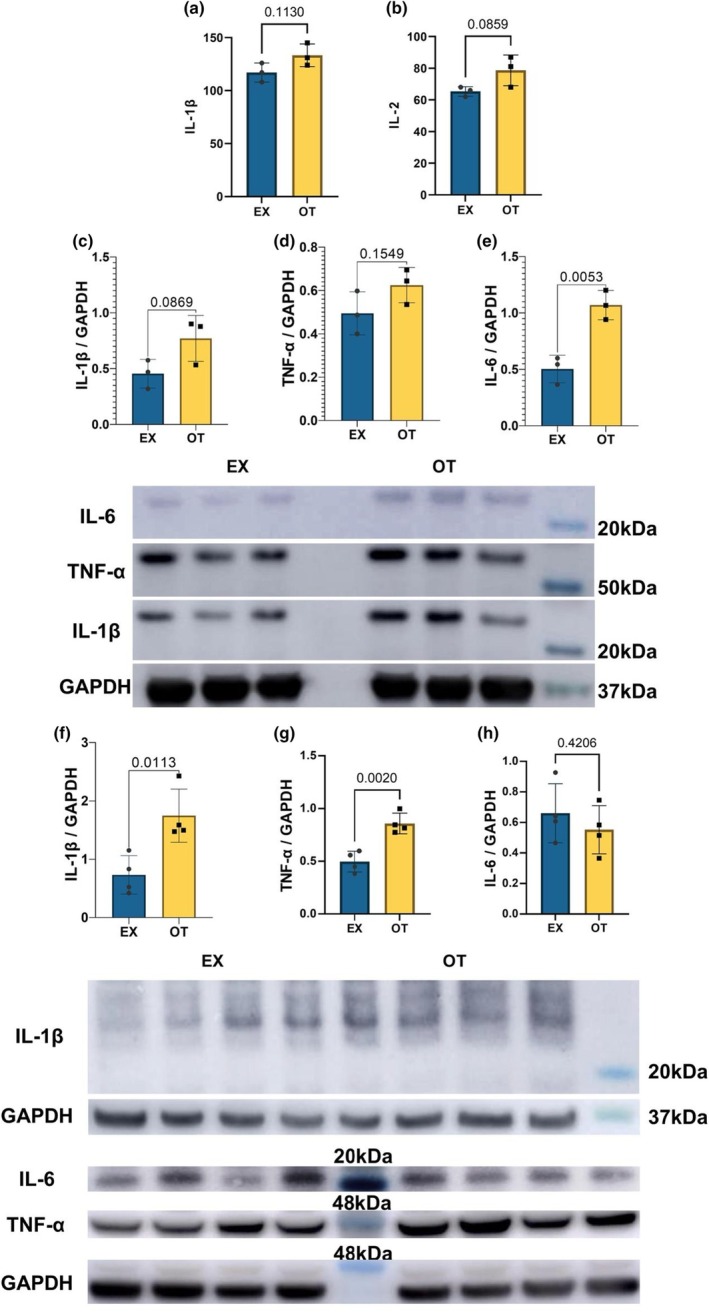
Inflammatory markers. (a) illustrates the blood plasma level of IL‐1β. (b) presents the blood plasma level of IL‐2. Western blot results of IL‐1β, TNF‐α, and IL‐6 in quadriceps are shown in (c–e). Western blot results of IL‐1β, TNF‐α, and IL‐6 in the hypothalamus are shown in (f–h). Data for (a–e) (*n* = 3), (f–h) (*n* = 4) are presented as mean ± SD. Unpaired *t*‐test for (a–h).

Blood plasma levels of IL‐1β and IL‐2 were higher in OT than EX, but not statistically significant (Figure 3a,b; *p* = 0.113, *p* = 0.0859). While levels of IL‐1β and TNF‐α were not changed in OT compared to EX in the quadriceps (Figure 3c,d; *p* = 0.0869, *p* = 0.1549), the level of IL‐6 was significantly higher in OT than EX in the quadriceps (Figure 3e; *p* < 0.01). Levels of IL‐1β and TNF‐α were significantly higher in OT than EX in the hypothalamus (Figure 3f,g; *p* < 0.05).

### 3.4 Validation of isolated CNS‐derived EVs

**FIGURE 4:**
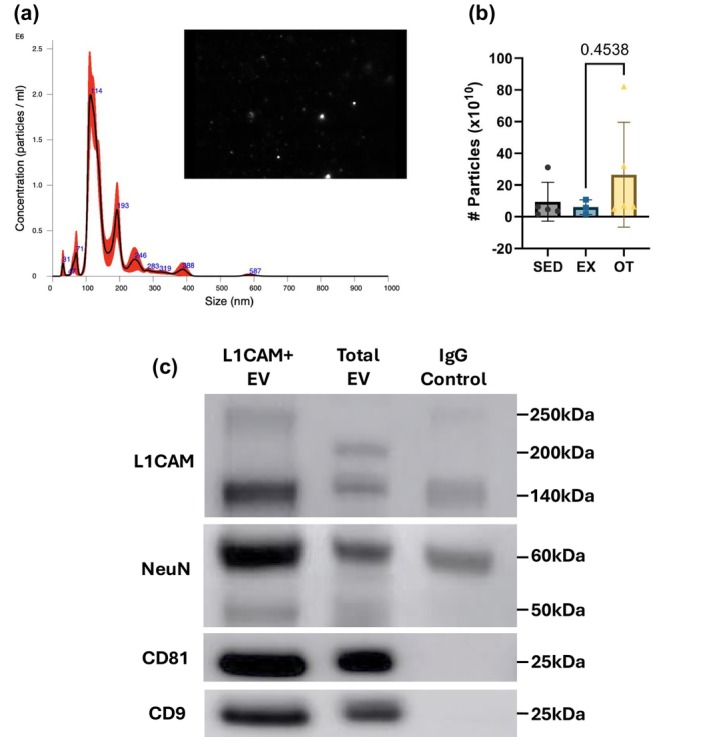
CNS‐derived EV validation. (a) presents particle concentration and size. (b) illustrates the number of particles per 1 mL of blood plasma. (c) shows the validation of CNS‐derived EVs through western blot results comparing total EVs and IgG isotype control of IP. EV markers CD81 and CD9, and CNS‐derived EV markers L1CAM and NeuN were validated by Western blot. Data for (a), (b) (SED, *n* = 5, EX, *n* = 3, and OT, *n* = 4) are presented as mean ± SD. Statistical analysis was conducted with one‐way ANOVA.

The average diameter of the particles was measured by NTA, an average of 211 nm (Figure 4a,b). In the Western blot (Figure 4c), immunoprecipitated EV with L1CAM (L1CAM + EV) exhibited higher levels of L1CAM, NeuN, and GFAP (Figure S1) than total EVs. The EV markers CD81 and CD9 showed equivalent signal intensities between L1CAM + EV and total EVs, while the IgG control showed a weaker signal (Figure 4c).

### 3.5 Effect of overtraining on CNS‐derived EV protein components

**FIGURE 5:**
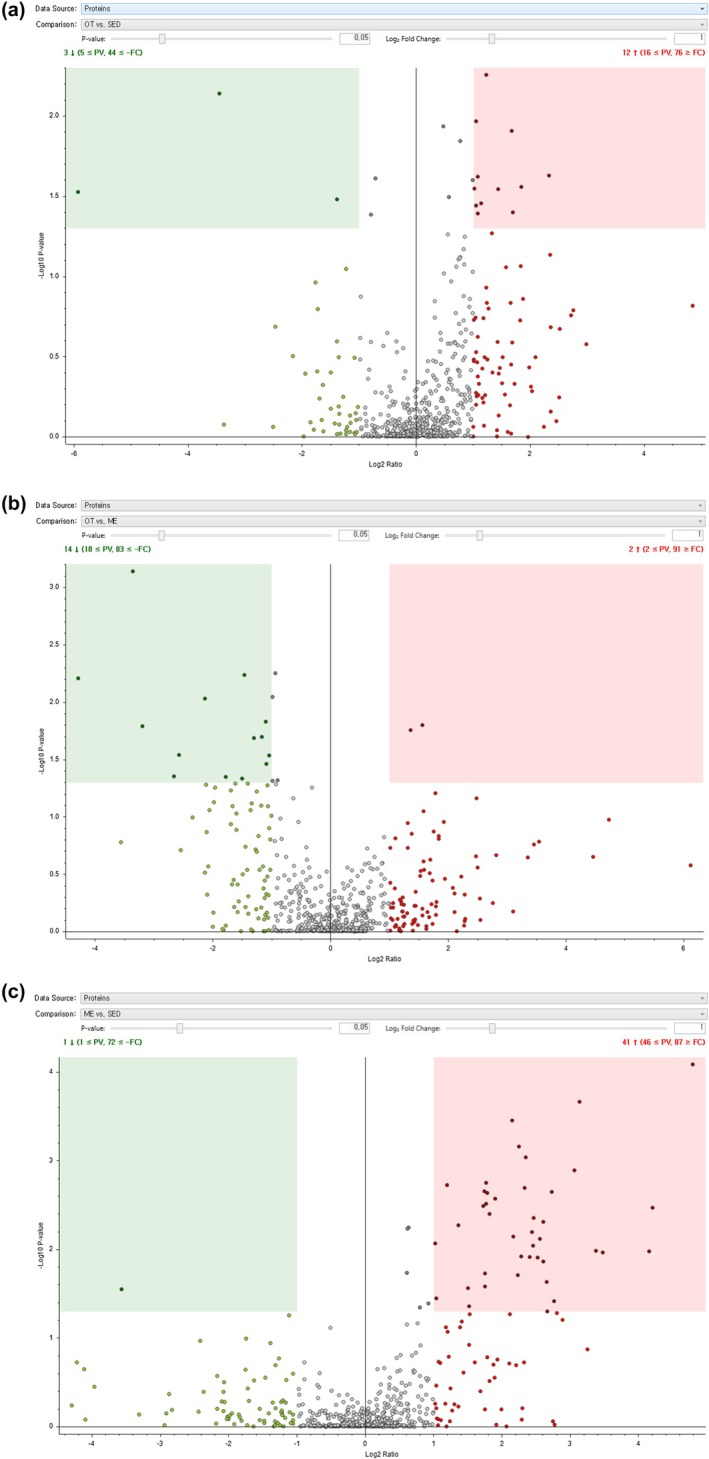
Proteomic analysis of CNS‐derived EVs. (a) presents the Volcano plot of CNS‐derived EVs of EX/SED. (b) shows the Volcano plot of CNS‐derived EV proteins of OT/EX. (c) displays the Volcano plot of CNS‐derived protein EVs of OT/SED. Data have been filtered by *p*‐value <0.05, Ratio ≥ |2|, (*n* = 3).

In comparison to EX, OT had 290 downregulated proteins and 268 upregulated proteins (Figure 5a), while EX had 313 upregulated proteins, and 245 downregulated proteins compared to SED (Figure 5b). After filtration, OT exhibited 14 upregulated proteins and two downregulated proteins compared to EX (Figure 5a), and 12 downregulated proteins and two upregulated proteins compared to SED (Figure 5b). EX had 41 upregulated proteins, and one downregulated protein compared to SED (Figure 5c). The statistically analyzed proteomic result data list is uploaded in (Table S1).

**FIGURE 6:**
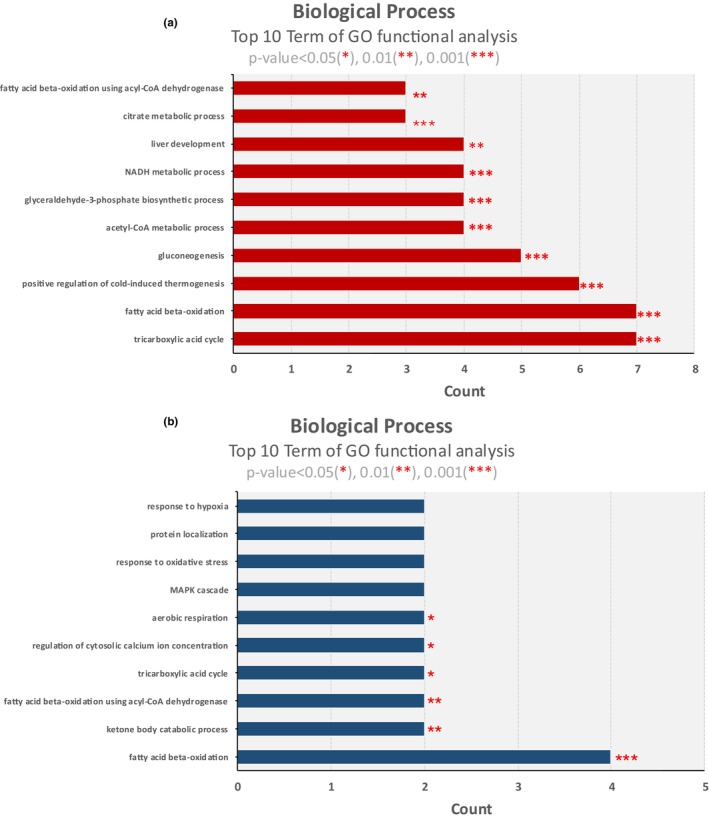
The enrichment analysis of biological processes. (a) presents biological process terms for up‐regulated proteins in CNS‐derived EVs of EX/SED. (b) presents biological process terms for down‐regulated proteins in CNS‐derived EVs of OT/EX. Gene ontology analysis was done using DAVID bioinformatics resources.

Gene Ontology analysis using DAVID Bioinformatics Resources parameters revealed insights into the roles of identified CNS‐derived EV proteins. Compared to SED, EX had 41 proteins upregulated during exercise, of which 7 proteins were involved in the tricarboxylic acid cycle, fatty acid beta‐oxidation, 6 proteins in positive regulation of cold‐induced thermogenesis, 5 proteins in gluconeogenesis, 4 proteins in acetyl‐CoA metabolic process, glyceraldehyde‐3‐phosphate biosynthetic process, NADH metabolic process, and liver development. Lastly, 3 proteins were involved in the citrate metabolic process and fatty acid beta‐oxidation using acyl‐CoA (Figure 6A).

In OT compared to EX, 14 proteins were downregulated by overtraining, of which 4 proteins were involved in fatty acid beta‐oxidation, and 2 proteins were involved in the ketone body catabolic process, fatty acid beta‐oxidation using acyl‐CoA, tricarboxylic acid cycle, regulation of cytosolic calcium ion concentration, and aerobic respiration. Two proteins were involved in the MAPK cascade, response to oxidative stress, protein localization, and response to hypoxia, but there was no statistical significance (Figure 6B).

## 4 DISCUSSION

In this study, we delved into the protein content of CNS‐derived EVs in an overtrained mouse model, identifying VCP and CAT as specific to overtraining. These proteins indicate stress and disease conditions in the CNS (Michiels et al., 1994).

Pereira et al., who investigated overtraining using an 8‐week downhill treadmill exercise mouse model (Pereira et al., 2012), reported a decrease in physical performance (Pereira et al., 2012) along with elevated levels of inflammatory cytokines in various organs, including the CNS and skeletal muscle (da Rocha et al., 2019). Building up to the downhill treadmill base overtraining protocol, our study observed a significant decrease in physical performance as well. Concurrently, the proinflammatory cytokine IL‐6 levels significantly increased in skeletal muscle. Notably, the IL‐1β and TNF‐α levels significantly increased in the hypothalamus, indicating a state of CNS fatigue in overtraining, consistent with previous research. Followed by biochemical response, OT scored lower in the nest building test, which presents motor deficits, as well as depression and anxiety levels. Several inflammatory cytokines did not show statistical significance. This may be because, although the mice were exposed to similar environments, the timing of nutritional supplementation or the time of exposure to each behavioral test may have influenced this inconsistency. In the following studies, it is necessary to prepare separate groups of mice for behavioral and biochemical analyses.

While various aspects of overtraining have been explored in past studies, none have employed EVs to investigate the overtraining syndrome. Given the symptomatology's connection to the CNS, we utilized CNS‐derived EVs to identify overtraining and potential biomarkers. Isolating CNS‐derived EVs followed the two‐step EV isolation method outlined by Mustapic (Mustapic et al., 2017). Similar to the Mustapic protocol, our validation of EV protein markers CD81 and CD9, as well as L1CAM, NeuN, and GFAP as CNS‐related markers through Western blot analysis, confirmed their higher signals compared to the total exosome lysate.

Proteomic analysis revealed upregulated fat metabolism‐related proteins in the EX compared to the SED. This finding indicates that EV contents are intricately linked to the energy demands of chronic exercise, supporting earlier research demonstrating the secretion and circulation of energy metabolism‐related molecules via small vesicles (Garcia et al., 2016; Yuana et al., 2013). In contrast to the comparison between the EX and SED, a notable downregulation of fat metabolism‐related proteins was observed in the OT compared to the EX. Such aberrant decreases in fat metabolism have been reported to be associated with neuromuscular junction denervation, mitochondrial dysfunction, excitotoxicity, impaired neuronal transport, cytoskeletal defects, inflammation, and reduced neurotransmitter release (Tracey et al., 2018). The roles of these proteins represent CNS fatigue and its relation to diseases affecting the peripheral nervous system. Based on the relationships of the shown proteins, they may contribute to decreased exercise capacity in the mice.

The proteomic analysis also unveiled a significant upregulation of both VCP and CAT in the OT compared to both the EX and SED (Table 1). VCP is implicated in apoptosis‐related pathways through endoplasmic reticulum stress during protein processing and is associated with various neuronal diseases, including Parkinson's disease, Alzheimer's disease, and amyotrophic lateral sclerosis (Kakizuka, 2008). CAT, an antioxidant enzyme ubiquitous in aerobic organisms, breaks down hydrogen peroxide into water and oxygen, with observed upregulation in organs experiencing oxidative stress (Michiels et al., 1994). Additionally, the roles of VCP and CAT in the context of overtraining remain unexplored. Given their critical associations with neurodegenerative diseases, these proteins could introduce novel elements in the field of overtraining research, potentially serving as biomarkers for overtraining and CNS fatigue, pending further investigation.

**TABLE 1:** CNS‐derived EV proteins specifically up‐regulated in OT.

Protein	Abundance ratio: (OT)/(EX)	Abundance ratio: (OT)/(SED)	Abundance ratio: (EX)/(SED)	*p*‐value: (OT)/(EX)	*p‐*value: (OT)/(SED)	*p*‐value: (EX)/(SED)
Valosin‐containing protein	2.955	3.239	1.096	0.016	0.04	0.72
Catalase	2.573	2.202	0.856	0.018	0.035	0.828

*Note*: It presents the abundance ratio and *p*‐value of specifically up‐regulated proteins between OT/EX, OT/SED, and EX/SED. Data were analyzed by the *Mus musculus* database from UniProt.

In conclusion, mice subjected to excessive exercise for 8 weeks exhibited symptoms indicative of overtraining, including elevated IL‐1β and TNF‐α levels in the hypothalamus. CNS‐derived EVs were isolated using a two‐step EV isolation method from blood plasma. These EVs from OT mice demonstrated heightened levels of CNS fatigue‐related proteins, namely, VCP and CAT. These proteins exhibited significant associations with CNS‐related diseases and pathways linked to cellular stress. While VCP and CAT showed upregulation due to overtraining, downregulated proteins were identified using DAVID Bioinformatics Resources, suggesting aberrant regulation of fat metabolism possibly contributing to fatigue in the brain and CNS. Although there are not many studies on EVs from overtrained athletes or individuals, certain miRNAs or proteins in EVs are being investigated as potential markers for monitoring CNS fatigue. The ability of EVs to cross the blood–brain barrier makes them particularly useful for detecting CNS dysfunction in peripheral fluids such as blood or cerebrospinal fluid. In future studies, it is necessary to establish a method to monitor the performance trends of athletes under extreme conditions and the tissue of origin of EVs in the blood. Therefore, we propose that CNS‐derived EVs may represent overtraining‐induced CNS fatigue. Furthermore, VCP and CAT emerge as potential biomarkers of CNS fatigue in overtraining syndrome.

This study has potential limitations. First, the cross‐sectional design restricts our ability to examine how CNS‐derived EVs and their associated proteins change over time in response to overtraining. For further research, applying a longitudinal approach with multiple time points before, during, and after the training period would offer a better understanding of the effect of how CNS‐derived EVs and the proteins respond to overtraining.

Second, the relatively small sample size (*n* = 24) across three groups may affect the statistical power and generalizability of the findings. Proteomic analysis was conducted on a subset of 3 samples per group due to technical constraints. This limitation was acknowledged, and data interpretation was performed with appropriate caution. Another limitation is that cytokine levels were assessed only in the EX and OT groups due to sample availability in the SED group. While this restricts comparison with baseline sedentary levels, the EX versus OT comparison remains informative and has precedent in previous studies focusing on training intensity effects. Additionally, although functional enrichment analysis using DAVID identified several GO biological process terms, the number of differentially expressed proteins per term was relatively small (mostly 2–4 proteins). This may have limited the biological interpretability and reduced the statistical power at the pathway level. A larger sample size in future studies would be beneficial for increasing statistical power and ensuring the robustness of the observed effects.

Third, our assessment of exercise capacity and fatigue relied on physical performance tests, including incremental loading, exhaustion tests, grip strength, and rotarod performance. Although these tests provide useful physiological data, they may not fully capture the complexity of overtraining syndrome. Adding behavioral and cognitive assessments, as well as measures of sleep patterns, would provide a better understanding of CNS fatigue.

Fourth, while we observed increased levels of proinflammatory cytokines, other potential confounding factors such as food intake, psychological stress, environment, and sex differences were not perfectly controlled. Given that these variables can impact cytokine levels and EV protein composition, future studies should control these factors for more specific effects on CNS‐derived EVs.

Lastly, the use of L1CAM as a marker for CNS‐derived EVs is the methodological limitation of this study. Although L1CAM is widely used to identify CNS‐derived EVs (Fiandaca et al., 2015; Mustapic et al., 2017; Nogueras‐Ortiz et al., 2024), on the other hand, its expression in non‐neuronal tissues raises concerns as well regarding specificity (Gomes & Witwer, 2022). While we included NeuN and GFAP as additional neuronal markers to support the presence of neuron‐derived EVs, it does not perfectly guarantee their origin.

To enhance the specificity of CNS‐derived EV identification, future research should consider additional CNS‐specific markers or employing more refined CNS‐derived EV isolation techniques. Furthermore, the uncertainty of the origination of EVs makes it important to acknowledge that the observed changes in protein content of EVs may reflect systemic physiological responses to overtraining. Addressing these challenges is essential for the understanding of both CNS‐derived EVs and the wider impact of exercise on circulating EVs.

By addressing these limitations, future research will expand our findings of CNS‐derived EVs in overtraining syndrome, leading to better monitoring and intervention strategies for athletes.

## AUTHOR CONTRIBUTIONS

Dong Heon Yi, Hyo Youl Moon and Adelino S R da Silva conceived and designed the research and analyzed data. Dong Heon Yi and Eun Seon Hwang performed experiments, interpreted the results, prepared figures, drafted the manuscript, edited and revised the manuscript, and approved the final version. Kang Eun Ko and Tae Yeon Kim collected quadricep samples. Ki Hoon Yook handled blood samples.

## FUNDING INFORMATION

This study was funded by the National Research Foundation (NRF) of Korea (NRF 2020R1C1C1006414, NRF‐2022R1I1A4053049, RS‐2025‐16066994) and was supported by the National Research Foundation of Korea (NRF) grant funded by the Korea Government (MSIT) (No. 2022R1A5A8019303).

## CONFLICT OF INTEREST STATEMENT

The authors declare no conflicts of interest, financial or otherwise.

## ETHICS STATEMENT

The animal study was reviewed and approved by the Institutional Animal Care and Use Committee of Seoul National University (SNU‐230321‐1).

## Supporting information


**Figure S1.** Additional validation for CNS derived EVs through western blot. GFAP was used as additional CNS derived marker. Common EV marker CD81 and CNS‐derived EV markers L1CAM were validated by western blot.

**Table S1.** Statistically analyzed full proteomic result data list. Abundance ratio data with abundance Ratio ≥ |2| and *p*‐value <0.05 are highlighted in red letter. *p*‐value of abundance ratio data with abundance Ratio ≥ |2| and *p*‐value <0.05 are highlighted in yellow colored cell.

## Data Availability

Data are available via ProteomeXchange with identifier PXD069257.

## References

[phy270640-bib-0001] Anderson, T. , Wideman, L. , Cadegiani, F. A. , & Kater, C. E. (2021). Effects of overtraining status on the cortisol awakening response—Endocrine and metabolic responses on overtraining syndrome (EROS‐CAR). International Journal of Sports Physiology and Performance, 16, 965–973. 10.1123/ijspp.2020-0205 33662935

[phy270640-bib-0002] Campbell, E. H. , Poudevigne, M. , McFarlane, S. , Dilworth, L. , & Irving, R. (2021). Evidence that sleep is an indicator of Overtraining during the competition phase of adolescent sprinters. Journal of Sports Medicine, 2021, 1–12. 10.1155/2021/6694547 PMC8041504 33884272

[phy270640-bib-0003] da Rocha, A. L. , Pinto, A. P. , Kohama, E. B. , Pauli, J. R. , de Moura, L. P. , Cintra, D. E. , Ropelle, E. R. , & da Silva, A. S. R. (2019). The proinflammatory effects of chronic excessive exercise. Cytokine, 119, 57–61. 30884427 10.1016/j.cyto.2019.02.016

[phy270640-bib-0004] Deacon, R. M. J. (2006). Assessing nest building in mice. Nature Protocols, 1, 1117–1119. 10.1038/nprot.2006.170 17406392

[phy270640-bib-0005] Fiandaca, M. S. , Kapogiannis, D. , Mapstone, M. , Boxer, A. , Eitan, E. , Schwartz, J. B. , Abner, E. L. , Petersen, R. C. , Federoff, H. J. , Miller, B. L. , & Goetzl, E. J. (2015). Identification of preclinical Alzheimer's disease by a profile of pathogenic proteins in neurally derived blood exosomes: A case‐control study. Alzheimer's & Dementia, 11, 600–607.e1. 10.1016/j.jalz.2014.06.008 PMC4329112 25130657

[phy270640-bib-0006] Garcia, N. A. , Moncayo‐Arlandi, J. , Sepulveda, P. , & Diez‐Juan, A. (2016). Cardiomyocyte exosomes regulate glycolytic flux in endothelium by direct transfer of GLUT transporters and glycolytic enzymes. Cardiovascular Research, 109, 397–408. 10.1093/cvr/cvv260 26609058

[phy270640-bib-0007] Gomes, D. E. , & Witwer, K. W. (2022). L1CAM‐associated extracellular vesicles: A systematic review of nomenclature, sources, separation, and characterization. Journal of Extracellular Biology, 1, e35. 10.1002/jex2.35 35492832 PMC9045013

[phy270640-bib-0008] Halson, S. L. , & Jeukendrup, A. E. (2004). Does Overtraining Exist? An Analysis of Overreaching and Overtraining Research. 10.2165/00007256-200434140-00003 15571428

[phy270640-bib-0009] Kakizuka, A. (2008). Roles of VCP in human neurodegenerative disorders. Biochemical Society Transactions, 36, 105–108. 10.1042/BST0360105 18208395

[phy270640-bib-0010] Kim, K. Y. , Shin, K. Y. , & Chang, K. A. (2021). Brain‐derived exosomal proteins as effective biomarkers for alzheimer's disease: A systematic review and meta‐analysis. Biomolecules, 11, 980. 34356604 10.3390/biom11070980 PMC8301985

[phy270640-bib-0026] Kuipers, H. , & Keizer, H. A. (1988). Overtraining in elite athletes: Review and directions for the future. Sports Medicine, 6, 79–92. 3062735 10.2165/00007256-198806020-00003

[phy270640-bib-0012] Meeusen, R. , Watson, P. , Hasegawa, H. , Roelands, B. , & Piacentini, M. F. (2007). Brain neurotransmitters in fatigue and overtraining. Applied Physiology, Nutrition and Metabolism, 32, 857–864. 10.1139/H07-080 18059610

[phy270640-bib-0013] Michiels, C. , Raes, O. T. , & Remacle, J. (1994). IMPORTANCE of CU/ZN‐sod for SE‐glutathione cell survival peroxidase, catalase, and against oxidative stress. Free Radical Biology and Medicine, 17, 235–248. 7982629 10.1016/0891-5849(94)90079-5

[phy270640-bib-0014] Mustapic, M. , Eitan, E. , Werner, J. K. , Berkowitz, S. T. , Lazaropoulos, M. P. , Tran, J. , Goetzl, E. J. , & Kapogiannis, D. (2017). Plasma extracellular vesicles enriched for neuronal origin: A potential window into brain pathologic processes. Frontiers in Neuroscience, 11, 278. 10.3389/fnins.2017.00278 28588440 PMC5439289

[phy270640-bib-0015] Nederveen, J. P. , Warnier, G. , Di Carlo, A. , Nilsson, M. I. , & Tarnopolsky, M. A. (2021). Extracellular vesicles and exosomes: Insights from exercise science. Frontiers in Physiology, 11, 604274. 33597890 10.3389/fphys.2020.604274 PMC7882633

[phy270640-bib-0016] Nogueras‐Ortiz, C. J. , Eren, E. , Yao, P. , Calzada, E. , Dunn, C. , Volpert, O. , Delgado‐Peraza, F. , Mustapic, M. , Lyashkov, A. , Rubio, F. J. , Vreones, M. , Cheng, L. , You, Y. , Hill, A. F. , Ikezu, T. , Eitan, E. , Goetzl, E. J. , & Kapogiannis, D. (2024). Single‐extracellular vesicle (EV) analyses validate the use of L1 cell adhesion molecule (L1CAM) as a reliable biomarker of neuron‐derived EVs. Journal of Extracellular Vesicles, 13, e12459. 10.1002/jev2.12459 38868956 PMC11170079

[phy270640-bib-0017] Pereira, B. C. , Filho, L. A. L. , Alves, G. F. , Pauli, J. R. , Ropelle, E. R. , Souza, C. T. , Cintra, D. E. , Saad, M. J. A. , & Silva, A. S. R. (2012). A new overtraining protocol for mice based on downhill running sessions. Clinical and Experimental Pharmacology & Physiology, 39, 793–798. 10.1111/j.1440-1681.2012.05728.x 22632058

[phy270640-bib-0018] Purvis, D. , Gonsalves, S. , & Deuster, P. A. (2010). Physiological and psychological fatigue in extreme conditions: Overtraining and elite athletes. PM & R: The Journal of Injury, Function, and Rehabilitation, 2, 442–450. 10.1016/j.pmrj.2010.03.025 20656626

[phy270640-bib-0025] Rocha, A. L. D. , Pereira, B. C. , Pauli, J. R. , Cintra, D. E. , de Souza, C. T. , Ropelle, E. R. , & R. da Silva, A. S. (2015). Downhill running‐based overtraining protocol improves hepatic insulin signaling pathway without concomitant decrease of inflammatory proteins. PloS one, 10, e0140020. 26445495 10.1371/journal.pone.0140020 PMC4596708

[phy270640-bib-0019] Si, X. , Tian, J. , Chen, Y. , Yan, Y. , Pu, J. , & Zhang, B. (2019). Central nervous system‐derived Exosomal alpha‐Synuclein in serum may Be a biomarker in Parkinson's disease. Neuroscience, 413, 308–316. 31102760 10.1016/j.neuroscience.2019.05.015

[phy270640-bib-0020] Tkach, M. , & Théry, C. (2016). Communication by extracellular vesicles: Where we are and where we need to go. Cell, 164, 1226–1232. 26967288 10.1016/j.cell.2016.01.043

[phy270640-bib-0021] Tracey, T. J. , Steyn, F. J. , Wolvetang, E. J. , & Ngo, S. T. (2018). Neuronal lipid metabolism: Multiple pathways driving functional outcomes in health and disease. Frontiers in Molecular Neuroscience, 11, 10. 29410613 10.3389/fnmol.2018.00010 PMC5787076

[phy270640-bib-0022] Whitham, M. , Parker, B. L. , Friedrichsen, M. , Hingst, J. R. , Hjorth, M. , Hughes, W. E. , Egan, C. L. , Cron, L. , Watt, K. I. , Kuchel, R. P. , Jayasooriah, N. , Estevez, E. , Petzold, T. , Suter, C. M. , Gregorevic, P. , Kiens, B. , Richter, E. A. , James, D. E. , Wojtaszewski, J. F. P. , & Febbraio, M. A. (2018). Extracellular vesicles provide a means for tissue crosstalk during exercise. Cell Metabolism, 27, 237–251. 10.1016/j.cmet.2017.12.001 29320704

[phy270640-bib-0023] Yuana, Y. , Sturk, A. , & Nieuwland, R. (2013). Extracellular vesicles in physiological and pathological conditions. Blood Reviews, 27, 31–39. 10.1016/j.blre.2012.12.002 23261067

[phy270640-bib-0024] Zisapel, N. (2001). Circadian rhythm sleep disorders. CNS Drugs, 15, 311–328. 10.2165/00023210-200115040-00005 11463135

