# Supplementary material for: Identifying overtraining biomarkers through proteomic analysis of extracellular vesicles derived from the central nervous system of male mice
Source: Physiol Rep. 2025 Nov 2;13(21):e70640. doi: 10.14814/phy2.70640 (PMC12580405; doi:10.14814/phy2.70640)
Supplement: Supplementary file 1 — Figure S1. Additional validation for CNS derived EVs through western blot. GFAP was used as additional CNS derived marker. Common EV marker CD81 and CNS‐derived EV markers L1CAM were validated by western blot. [file PHY2-13-e70640-s002.pdf]

Figure S1.

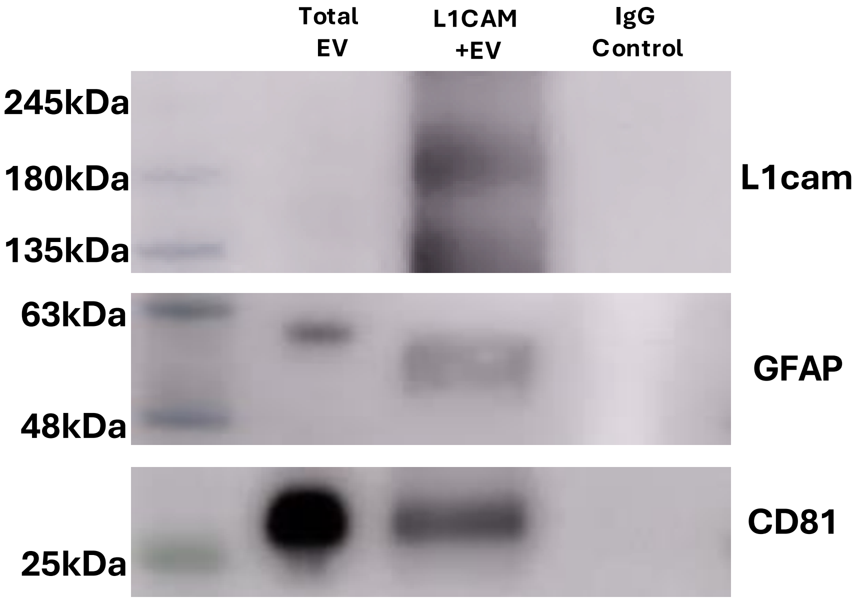

Figure S1. Additional validation for CNS derived EVs through western blot. GFAP was used as additional CNS derived marker. Common EV marker CD81 and CNS-derived EV markers L1CAM were validated by western blot.
